# Inflammatory Biomarkers and Lipid Parameters May Predict an Increased Risk for Atrial Arrhythmias in Patients with Systemic Sclerosis

**DOI:** 10.3390/biomedicines13010220

**Published:** 2025-01-16

**Authors:** Veronika Sebestyén, Dóra Ujvárosy, Balázs Ratku, Hajnalka Lőrincz, Sára Csiha, Dóra Tari, Gyöngyike Majai, Sándor Somodi, Gabriella Szűcs, Mariann Harangi, Zoltán Szabó

**Affiliations:** 1 Department of Emergency Medicine, Faculty of Medicine, University of Debrecen, 4032 Debrecen, Hungary; sebestyen.veronika@med.unideb.hu (V.S.); ujvarosy.dora@med.unideb.hu (D.U.); ratkubalazs@gmail.com (B.R.); somodi@belklinika.com (S.S.); 2 Doctoral School of Health Sciences, University of Debrecen, 4032 Debrecen, Hungary; 3 Faculty of Health Sciences, Institute of Health Studies, University of Debrecen, 4032 Debrecen, Hungary; harangi@belklinika.com; 4 Division of Metabolism, Department of Internal Medicine, Faculty of Medicine, University of Debrecen, 4032 Debrecen, Hungary; lorincz_hajnalka@belklinika.com (H.L.); csiha.sara@med.unideb.hu (S.C.); 5 Department of Rheumatology, Faculty of Medicine, University of Debrecen, 4032 Debrecen, Hungary; dora.tari05@gmail.com (D.T.); szucsgpafi@gmail.com (G.S.); 6 Division of Clinical Immunology, Department of Internal Medicine, Faculty of Medicine, University of Debrecen, 4032 Debrecen, Hungary; mgyongyi@med.unideb.hu

**Keywords:** systemic sclerosis, atrial arrhythmias, inflammation, leptin, oxLDL, LDL and HDL subfractions, electrocardiography

## Abstract

Background/Objectives: Autoimmune inflammation enhances the electrical instability of the atrial myocardium in patients with systemic sclerosis (SSc); thus, atrial arrhythmia risk is increased, which might be predicted by evaluating the P wave interval and dispersion of a 12-lead surface electrocardiogram (ECG). Methods: We examined 26 SSc patients and 36 healthy controls and measured the P wave interval and P wave dispersion of the 12-lead surface ECG in each patient. Furthermore, echocardiography and 24-h Holter ECG were performed and levels of inflammatory laboratory parameters, including serum progranulin (PGRN), sVCAM-1, sICAM-1, leptin and C-reactive protein (CRP), were determined. Lipid parameters, such as Apo A-I, LDL-cholesterol (LDL-C), oxidized LDL (oxLDL) and the LDL and HDL subfractions were also evaluated. Results: The P wave interval showed a significant positive correlation with the levels of Apo A-I, LDL-C, CRP, sVCAM-1, sICAM-1 and leptin. The oxLDL level correlated positively with P wave dispersion. Of note, significant positive correlation was also found between the large HDL percentage and the P wave interval. Conclusions: Our results suggest that PGRN, sVCAM-1, sICAM-1, leptin, CRP, LDL-C and oxLDL, along with LDL and HDL subfractions, might have a role in atrial arrhythmogenesis in patients with SSc.

## 1. Introduction

Systemic sclerosis is an autoimmune condition, which is characterized by fibrotic tissue deposition in the skin and internal organs, endothelial dysfunction and the presence of autoimmune inflammation [1]. The disease has a female predominance with an overall female to male ratio of 3:1 [2]. Systemic sclerosis (SSc) is associated with structural inhomogeneity and patchy fibrosis of the myocardium, which can result in anisotropy and electrical instability, leading to pulse conduction disorders and heart rhythm disturbances [3,4]. Furthermore, increased arrhythmia risk may develop secondary to reversible ischemic changes due to coronary vasoconstriction, the appearance of circulating b-adrenergic receptor autoantibodies and intracardiac pressure overload resulting from pulmonary arterial hypertension [5]. Based on previous studies, scleroderma heart involvement (SHI) is associated with worse prognosis as well as higher mortality [6,7]. Importantly, 6% of SSc-related cardiovascular mortality is arrhythmia-related mortality [8]. Supraventricular arrhythmias can be present in 32–66% of scleroderma patients, where supraventricular premature beats are the most common entities (2.6–90%) [5,9]. Moreover, atrial fibrillation (AF) occurs in 1.8–36.7% of SSc patients [9]. Fairley et al. found that in the phase of subclinical cardiac manifestations, the incidence of AF is not higher in SSc patients compared to the control population [10]. On the contrary, the presence of atrial and supraventricular arrhythmias is 7.1 times higher in SSc patients with advanced cardiac manifestation compared to healthy subjects [10]. In-hospital mortality of SSc patients with atrial fibrillation has been shown to be three times higher compared to patients with AF without coexisting scleroderma [11]. Interestingly, in SSc patients AF can develop regardless of the presence of the classic cardiovascular risk factors, such as dyslipidemia, obesity or hypertension [11]. Of note, systemic sclerosis patients have an increased risk for conduction disorders and rhythm disturbances compared to nonsystemic sclerosis patients even in the early phase of the disease [12].

Inflammation plays an important role in the genesis of electrical and structural remodeling of the atrial myocardium in patients with SSc [8]. The P wave interval (P int), maximal P wave interval (P max) and P dispersion (Pd) of a 12-lead surface ECG have been confirmed to indicate the electrical inhomogeneity of the atrial myocardium. The lengthening of the P max and Pd have also been described in patients with SSc [13]. Evaluating the data of 23 patients with SSc, Wokhlu et al. have demonstrated that P wave amplitude in lead II of the electrocardiogram correlated with pulmonary arterial pressure [14]. In an investigation conducted by Sharifkazemi et al. the left atrial volume, the left atrial area and the diameter of the inferior vena cava were found to be higher in scleroderma patients compared to controls [15,16]. Durmus et al. found reduced right ventricular strain parameters, tricuspid annular plane systolic excursion (TAPSE) and right atrial reservoir and conduit strain parameters in SSc patients without the presence of pulmonary arterial hypertension [17]. In another echocardiographic study by Aktoz et al., the value of the left ventricular E wave correlated with parameters describing the left atrial electrical and mechanical delay, suggesting the usefulness of these parameters in atrial fibrillation risk stratification in this patient population [13]. In addition to the aforementioned methods, cardiac magnetic resonance (CMR) has proven to be a very useful method in the assessment of early heart involvement in scleroderma patients [7].

The gold standard laboratory markers for the detection of scleroderma heart involvement (SHI) are high sensitivity troponin (HScTnT) and N-terminal prohormone of b-type natriuretic peptide (NT-proBNP), of which HScTnT is more specific for SHI and can be elevated even in the subclinical phase of the disease [18]. In the case of SSc patients, the presence of dyslipidemia contributes to an adverse cardiovascular risk profile. Ferraz-Amaro et al. examined 73 SSc patients and 115 controls, and found lower high-density lipoprotein cholesterol (HDL-C), low-density lipoprotein cholesterol (LDL-C) and apolipoprotein A-I (Apo A-I) levels and higher lipoprotein (a) (Lp (a)), triglyceride, Apo B/A-I ratio and atherogeneity index (total cholesterol/HDL-C) in the scleroderma group [19]. Kotyla et al. demonstrated higher LDL-C and total cholesterol levels, while Lippi et al. showed higher C-reactive protein (CRP) and Lp (a) levels in SSc patients compared to controls [20,21]. Based on the so-called lipid paradox, which has been described in several autoimmune diseases, in untreated inflammatory processes the serum total cholesterol and LDL-C levels are lower. This phenomenon is considered the consequence of the lipid lowering effect of the inflammatory response itself [22]. In the development of atherosclerotic plaques, oxidized LDL (oxLDL) is known as a crucial risk factor. OxLDL together with immunoreactive lymphocytes presumably plays a role in the ongoing immune processes affecting the arterial wall [23]. The serum levels of intercellular adhesion molecule 1 (ICAM-1) and vascular cell adhesion molecule 1 (VCAM-1) correlate with disease severity in SSc. These may contribute to the development of skin fibrosis and are able to aggravate the harmful processes [24,25]. An organokine-like molecule called progranulin (PGRN) also takes part in the ongoing inflammation due to the modulation of the activity of the tumor necrosis factor α (TNF-α) signaling pathway. Cardioprotective effects of PGRN have been demonstrated in numerous studies, although hyperprogranulinemia can also be associated with enhanced skin fibrosis in SSc patients [26]. Furthermore, leptin can modulate inflammatory responses, since it can upregulate the production of proinflammatory cytokines (e.g., IL-6, IL-12, TNF-α) and may lead to the activation of immune cells such as natural killer- and T helper 1-cells and macrophages [27,28]. Previous studies showed contradictory results regarding the leptin levels in SSc patients [27]. Interestingly, lower leptin levels were linked to active disease [29].

Scleroderma has poor prognosis due to severe organ involvements [30]. Nowadays, the major causes of mortality in this population are SHI, pulmonary arterial hypertension and their consequences [4]. As atrial arrhythmias frequently occur in SSc patients [10], the recognition of patients with elevated arrhythmia risk is particularly important. The aim of our study was to determine the potential risk markers of atrial arrhythmias and examine the correlations between clinical markers and laboratory parameters in patients with SSc.

## 2. Materials and Methods

### 2.1. Patients

The study population was made up of 26 SSc patients and 36 healthy controls, who were between 18 and 70 years of age. Patients with any type of diabetes mellitus, structural heart diseases, atrial fibrillation or the history of any other kind of arrhythmia events were excluded from the study. Patients with hypothyroidism could enter the study if they were in a stable euthyroid state. Inclusion and exclusion criteria of the examined SSc population are summarized in Table 1.

Written informed consent was obtained from all participants before study entry. The Medical Research Council and the Hungarian Ministry of Human Resources (Deputy State Secretary for National Chief Medical Officer, Department of Health Administration) approved our study protocol (number of approvals: 21325-2-1/2017/EKU, 11920-2/2017/EÜIG and 19927-1/2018/EKU). We performed our investigations according to the Declaration of Helsinki.

### 2.2. Echocardiography

Transthoracic echocardiography was performed by using a Philips HDI ATL 5000 imaging system (Philips ATL ULTRASOUND INC., Washington, DC, USA) and a 3.5 MHz transducer (Acuson Sequoia C 256 Mountain View, Siemens Medical Solutions USA, Mountain View, CA, USA). During our examinations, we used 2D, M-mode, pulsatile and continuous wave Doppler techniques to determine the parameters describing the systolic and diastolic function of the right and left sides of the heart. In addition to the left atrial diameter, we measured the value of the left atrial area, the diameter of the inferior vena cava, the right ventricular systolic pressure, the TAPSE indicating the right ventricular systolic function and the mitral E/A and the left ventricular filling pressure (E/e’) by means of the tissue Doppler technique.

### 2.3. Electrocardiography

We gained a standard 12-lead surface electrocardiogram (ECG) and a digital ECG from every patient. Digital ECG recordings were obtained and analyzed with the use of CardioSys Plus ECG analyzing hardware and software (MDE GmBH, Walldorf, Germany). During the ECG analysis, we determined the P wave interval (P int), maximal P wave interval (P max) and P wave dispersion (Pd) with the help of CardioSys software (CardioSys CA-12, Logirex Kft., Budapest, Hungary) [31,32]. The program calculates the P wave interval as an average of five consecutive P waves in every lead. The maximal P wave interval is determined as the longest P wave in the given lead. In the results section, we highlight the P interval (P int) and maximal P interval (P max) values determined from the analysis of standard lead II, because these are the most reliable for the comparison of different ECG recordings based on previous studies [33]. Furthermore, P wave dispersion has been calculated as the difference between the longest and the shortest P wave intervals in the 12 leads of the electrocardiogram [34,35]. Moreover, 24-h Holter ECG monitoring was performed with three-channel CardioMera ECG-Holter monitors (MediTech Kft, Budapest, Hungary) using CardioVisions software (CardioVisions 1.30.2, MediTech Kft, Budapest, Hungary), where average heart rate, average RR cycle length, time- and frequency-based heart rate variability, arrhythmia events and conduction disorders were evaluated.

### 2.4. Laboratory Parameters

Routine laboratory parameters were determined from fresh sera according to the standard laboratory protocols at the Department of Laboratory Medicine of the University of Debrecen. Besides serum electrolyte levels, the scleroderma autoantibody profile, the cardiac troponin T, creatine-kinase, NT-proBNP levels, and the thyroid and kidney function, lipid profile, CRP level and blood cell counts of the patients were also measured.

The soluble form of VCAM-1 (sVCAM-1), the soluble form of ICAM-1 (sICAM-1) and serum PGRN, leptin and oxLDL levels were determined with the ELISA method at the Research Laboratory of the Division of Metabolism, Department of Internal Medicine, Faculty of Medicine at the University of Debrecen (human PGRN ELISA kit: Cat. No. RMEE103R, BioVendor, Brno, Czech Republic; human sVCAM-1, sICAM-1 and leptin ELISA kits: Cat. No. DVC00, DCD540 and DLP00, R&D Systems Europe Ltd., Abington, UK; oxLDL: Cat. No. 10-1143-01, Mercodia AB, Uppsala, Sweden). LDL and HDL subfractions were determined based on an electrophoresis on polyacrylamide gel, termed Lipoprint^®^ (Quantimetrix Corp., Redondo Beach, CA, USA). This method distributes lipoprotein subfractions based on their size. During the LDL lipoprotein test (Quantimetrix Corp., Cat. No. 48-7002, Redondo Beach, CA, USA), the percentages of very low-density lipoprotein (VLDL), intermediate-density lipoprotein (IDL; MID-A, -B and –C) and up to seven LDL subfractions were measured. The small LDL percentage was calculated from the mean ratios of LDL 3–7 subfractions, while the large LDL percentage was defined as the average of the ratio of LDL 1–2 subfractions. In addition, we evaluated ten HDL subfractions during the HDL lipoprotein test (Quantimetrix Corp., Cat. No. 48-9002, Redondo Beach, CA, USA). The large HDL was calculated from the average of HDL 1–3 subfractions, the intermediate HDL group from HDL 4–7 subfractions, and the small HDL from HDL 8–10 subfractions. With the help of Lipoware Image SXM v.1.82 Software (Quantimetrix Corp., Redondo Beach, CA, USA), we also calculated the absolute quantities of these parameters using the actual total cholesterol and HDL-C values [36]. The mean LDL size was also determined with Lipoware software. Liposure normal control (provided by Quantimetrix corp., Cat. No. 48-7060, Redondo Beach, CA, USA) was used as a standard in each electrophoresis.

### 2.5. Statistical Analysis

Statistical analysis was performed by using GraphPad Prism software (version 9.1.1. (225), GraphPad Software, San Diego, CA, USA). Basic descriptive statistics (arithmetic mean, median, 90 and 95% confidence intervals and different quartile values) are described. The normality of the data was checked by applying the Kolmogorov–Smirnov test. To investigate the possible significant difference between the statistical variables, we used ordinary one-way ANOVA (analysis of variance). Pairwise comparisons were made with *t*-tests in the case of normal and Mann–Whitney tests in the case of non-normal distribution. Welch correction was added in case of significant differences between the calculated variances. The difference between categorical variables was calculated with the Chi-squared test. Correlations between the variables were analyzed using Pearson’s test in the case of normal distribution and Spearman’s rank test in the case of non-normally distributed data. The level of statistical significance was *p* < 0.05.

## 3. Results

### 3.1. Clinical Parameters

Clinical parameters of the enrolled 26 SSc patients and 36 age-, gender- and BMI-matched healthy controls are summarized in Table 2. The ratio of patients who took statin regularly did not differ significantly between the subgroups. Twelve scleroderma patients suffered from the limited cutaneous form, while fourteen suffered from the diffuse cutaneous form of the disease. The average time elapsed from the diagnosis was 4.1 years, while 6.5 years passed before the detection of the first clinical symptoms on average.

### 3.2. Electrocardiographic Findings

**Figure 1:**
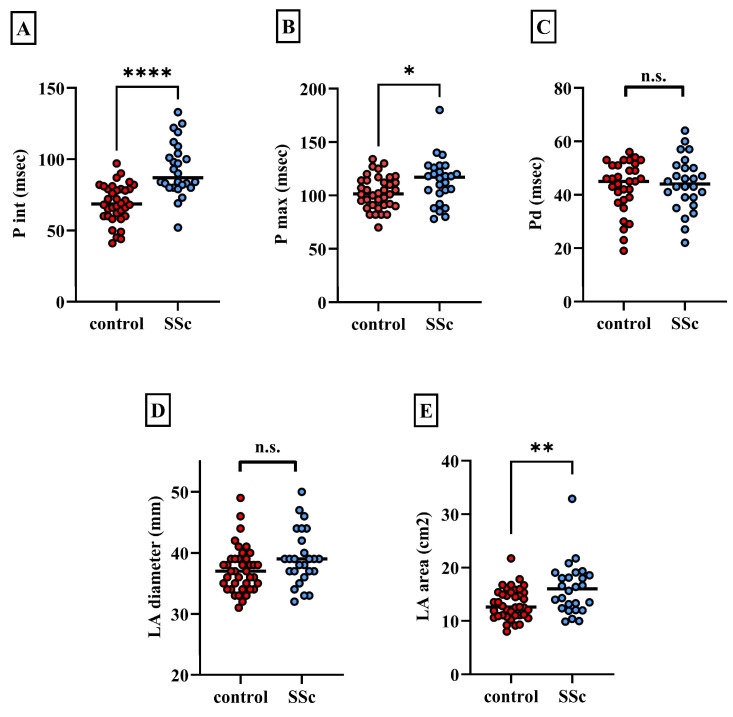
(**A**–**C**): Pairwise comparisons of atrial arrhythmia risk markers between the control and SSc groups. (**D**,**E**): Echocardiographic parameters describing the size of the left atrium in the control and SSc groups. LA: left atrium, P int: P wave interval, P max: maximal P wave interval, Pd: P wave dispersion. * *p* < 0.05; ** *p* < 0.01; **** *p* < 0.0001; n.s.: not significant.

Regarding the ECG markers, we found that the P wave interval (P int) and the maximal P wave interval (P max) significantly lengthened in the case of SSc patients compared to healthy controls (P int: 69.11 ± 13.4 msec vs. 92.92 ± 18.2 msec, *p* < 0.0001; P max: 102.8 ± 15.3 msec vs. 113.5 ± 20.9 msec, *p* < 0.05). Interestingly, P wave dispersion (Pd), characterizing the spatial inhomogeneity of the atrial myocardium, was not significantly different between the two groups of patients (43.14 ± 9.6 msec vs. 44.08 ± 10 msec, *p* = 0.71) (Figure 1).

In our study, 24-h Holter ECG examinations were performed. There was no significant difference in the ratio of supraventricular premature beats between the studied groups. In the control group, we detected mainly sinus tachycardia episodes. Importantly, in the SSc group we observed paroxysmal atrial fibrillation in four patients (an average of 3.2 episodes/person with an average duration of 1.5 h during the 24-h detection period). In two patients, we registered paroxysmal supraventricular tachycardia (AVNRT) episodes (an average of 2.6 arrhythmia events/person, with a mean duration of 5.3 min).

### 3.3. Echocardiographic Results

Regarding echocardiographic parameters, we did not find a significant difference between the left atrial diameter values (37.2 ± 3.78 mm vs. 39.1 ± 4.5 mm, *p* = 0.075), while the left atrial area was significantly higher in SSc patients compared to controls (13.2 ± 2.9 cm^2^ vs. 16.1 ± 4.6 cm^2^, *p* < 0.01, Figure 1). Further electrocardiographic and echocardiographic parameters are summarized in Table 3. Additional statistical data about electrocardiographic and echocardiographic parameters are available as a Supplementary Material in Table S1.

Both the left atrial area and left atrial diameter showed a positive correlation with the P wave interval, but it only reached the level of significance in the case of the left atrial diameter (r = 0.35, *p* = 0.07; r = 0.47, *p* < 0.05, respectively).

### 3.4. Laboratory Results

Apo A-I levels were lower in scleroderma patients than in the controls (1.41 ± 0.2 g/L vs. 1.52 ± 0.22 g/L, *p* < 0.05), but the Apo-B and Lp (a) levels were not significantly different between the groups (1.06 ± 0.25 g/L vs. 0.99 ± 0.25, *p* = 0.15; 65 (35–138) mg/L vs. 64.5 (32.25–112.3, *p* = 0.24) mg/L, respectively, Table 4). There was no significant difference between the scleroderma and the control groups regarding the LDL/HDL-cholesterol ratio, Apo B/Apo A-I ratio and atherogeneity index. The oxLDL level was lower in SSc patients than in the controls, but this difference did not turn out to be statistically significant (67.3 ± 18.72 U/L vs. 74.54 ± 18.1 U/L, *p* = 0.28, Table 4).

### 3.5. Correlations Between Parameters

**Figure 2:**
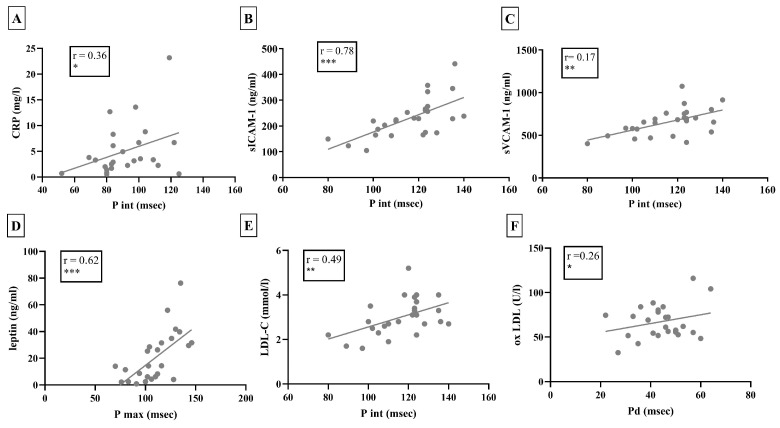
Correlations of the atrial arrhythmia risk markers with the different examined parameters in SSc patients. (**A**): Correlation between the CRP and P wave interval. (**B**,**C**): Correlations between the levels of adhesion molecules and P wave interval. (**D**–**F**): Correlations of different lipid parameters and adipokines with the atrial arrhythmia risk markers. CRP: C-reactive protein, sICAM-1: soluble ICAM-1, sVCAM-1: soluble VCAM-1, Apo A-I: apolipoprotein A-1, LDL-C: low-density lipoprotein cholesterol, oxLDL: oxidized low-density lipoprotein, P int: P wave interval, P max: maximal P wave interval, Pd: P wave dispersion. * *p* < 0.05; ** *p* < 0.01; *** *p* < 0.001.

Among laboratory parameters, the NT-proBNP and CRP levels showed a significant positive correlation with the P wave interval (r = 0.24, *p* < 0.05; r = 0.36, *p* < 0.05, respectively). Serum PGRN levels did not show significant correlations with P int, while the sICAM-1 and the sVCAM-1 levels correlated positively with P int (PGRN: r = −0.003, *p* = 0.99; sICAM-1: r = 0.78, *p* < 0.001; sVCAM-1: r = 0.17, *p* < 0.01). During the investigation of the correlations between lipid parameters and atrial arrhythmia risk markers, we found a significant positive correlation between the P wave interval and LDL-C levels (r = 0.49, *p* < 0.01, Figure 2). Furthermore, the oxLDL level correlated positively with the P wave dispersion among scleroderma patients (r = 0.26, *p* < 0.05, Figure 2). The serum leptin level also showed a significant positive correlation with the value of the maximal P wave interval (r = 0.62, *p* < 0.001, Figure 2).

During the Lipoprint analyses we found that the IDL percentage was significantly higher in scleroderma patients compared to the control group, while there was no significant difference regarding VLDL percentage (Table 4). Interestingly, the ratio of large LDL subfractions was significantly lower in scleroderma patients (31.92 ± 4.45% vs. 28.25 ± 5.4; *p* > 0.01). Small LDL subfractions were not different between the scleroderma and the control groups (Table 4). In our study, we found a tendency to lower mean LDL size in the controls compared to SSc patients, but a statistically significant difference could not be revealed (27.04 ± 0.03 vs. 26.81 ± 0.05, *p* = 0.06, Table 4). The mean large HDL percentage was higher, while the intermediate and small HDL percentages were lower in scleroderma patients compared to the controls. Only the intermediate HDL-C showed a statistically significant difference (50.54 ± 3.87% vs. 48.56 ± 4%; *p* < 0.05) (Table 4). Furthermore, sVCAM-1 and serum PGRN levels showed significant negative correlations with the large HDL percentage (r= −0.41, *p* < 0.05; r = −0.35, *p* < 0.05, respectively). On the contrary, the small HDL percentage correlated positively with the serum PGRN levels in SSc patients (r = 0.42, *p* < 0.05).

**Figure 3:**
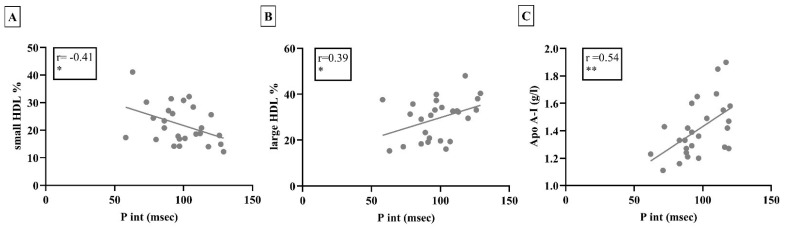
(**A**): The P wave interval correlated negatively with the small HDL percentage. (**B**): The P wave interval showed a positive correlation with the large HDL percentage. (**C**): The Apo A-I level was found to be positively correlated with the P wave interval. HDL: high-density lipoprotein, P int: P wave interval, Apo A-I: apolipoprotein A-I, * *p* < 0.05; ** *p* < 0.01.

We also investigated the potential associations between electrocardiographic parameters and lipid subfractions. The P wave interval showed significant negative correlation with the small HDL percentage, whilst it was correlated positively with the large HDL percentage (r = −0.41, *p* = 0.037; r = 0.39, *p* = 0.049, respectively, Figure 3). Interestingly, a significant positive correlation was found between the P wave interval and the Apo-A-I levels (r = 0.54, *p* < 0.01; Figure 3).

Furthermore, the P wave dispersion showed a positive correlation with the VLDL percentage (r = 0.28, *p* = 0.03). In addition, we found a significant negative correlation between the IDL percentage and the P wave interval (r = −0.43, *p* = 0.03). These correlations were not observed in the control group.

## 4. Discussion

Due to the electrical inhomogeneity of the myocardial tissue caused by ongoing fibrotic and inflammatory processes, we can expect an increased risk for atrial arrhythmias in patients with scleroderma. This is supported by international literature and confirmed by our results [13], showing increased P wave interval and P wave dispersion in SSc patients compared to the controls. Based on several studies, SSc patients demonstrate frequent supraventricular premature beats during 24-h Holter ECG recordings [37,38]. In our investigation, atrial premature beats were also detected during Holter electrocardiography; however, no significant difference was found between the SSc group and the controls. Even though our SSc patients had no previous history of arrhythmia events, we detected sinus tachycardia episodes (15 episodes/person in average), paroxysmal supraventricular tachycardia (AVNRT) events and paroxysmal atrial fibrillation episodes, proving the increased risk for atrial arrhythmias in patients with scleroderma.

Left atrial dilation may lead to higher risk of supraventricular arrhythmias. Echocardiography is one of the most useful methods to describe the size of the left atrium. Left atrial diameter determined from the parasternal long axis view with the help of 2D and M-mode examinations is an accurate, reproducible parameter, which is suitable for statistical analyses [39,40]. However, sometimes the left atrium has a spatial asymmetry, which negatively affects the sensitivity of this measurement [41]. Therefore, determining the left atrial area can provide a more accurate and reproducible estimation. In our study, we found that both the left atrial diameter and the left atrial area correlated positively with the P wave interval, suggesting a role of the left atrial size in the genesis of atrial rhythm disturbances.

Scleroderma patients may have an elevated serum CRP level compared to healthy controls [21], which has also been confirmed in our present study. Additionally, we found a positive correlation between the CRP levels and the P wave interval, which highlights the unfavorable effect of inflammatory processes on atrial arrhythmia susceptibility.

In our study, lower Apo-A-I levels were detected in the scleroderma group compared to the control, where Apo-A-I levels were shown to be within the normal range. In a previous study, low Apo-A-I levels showed a significant correlation with the QTc interval in SSc patients, showing an increased risk for ventricular arrhythmia susceptibility [38]. In another study by Zhong et al., low Apo-A-I levels increased the risk for atrial fibrillation in those individuals who did not take statins regularly [42]. On the contrary, we found that the ratio of large HDL and the Apo-A-I level showed a significant positive correlation with the P wave interval, suggesting that Apo-A-I and large HDL levels may also be associated with an increased risk for atrial arrhythmias in this population. Apo A-I is one of the most important structural proteins of the HDL molecule, from which small HDL particles usually contain 2 molecules, while the larger HDL particles may contain as many as 5–7 molecules, so the large HDL has a higher Apo A-I content [43]. Previous studies showed significant differences in the quality and quantity of further HDL-associated proteins between the HDL subfractions. The human paraoxonase 1 and 3, with known antioxidant activity, and the antiatherogenic apolipoprotein M and sphingosine-1-phosphate can be detected mainly on the small HDL particles, while the proatherogenic apolipoprotein B and α-2 macroglobulin are bound to the large HDL particles [44,45]. These differences in the protein composition between the subfractions may explain our observation according to which the large HDL subfractions and the Apo A-I correlate positively with arrhythmia markers.

The lipid paradox described in autoimmune disease [22] could also be detected in our scleroderma patients, who demonstrated lower LDL-C levels compared to the controls. This observation may be the consequence of the lipid level lowering effect of the ongoing inflammatory disease. Interestingly, decreased LDL-C levels were found to be associated with the development of atrial fibrillation in the general population, which supports the observation that the use of statins does not decrease the risk for atrial fibrillation [46]. Furthermore, we detected a negative correlation between the LDL-C level and the P wave interval in the control group (r = −0.35; *p* = 0.056). Conversely, LDL-C levels correlated positively with the P wave interval in SSc patients. This may suggest that the autoimmune inflammatory process can influence the effects of lipid levels on arrhythmia risk. Moreover, we conclude that the lipid lowering therapy and lipid goal attainment can be more important in the reduction in the atrial arrhythmia risk in SSc patients than in healthy subjects.

Previously, our research group demonstrated that LDL-C, TG and Apo B levels correlated positively with the QT dispersion, which indicates elevated ventricular arrhythmia risk [47]. Consequently, dyslipidemia may increase the ventricular arrhythmia risk presumably due to its effect on the cell membrane function and composition [47].

In our study, during the evaluation of LDL and HDL subfractions, we found significantly lower large LDL percentages and intermediate HDL percentages in scleroderma patients than in healthy controls. This difference in the lipid profile can play a role in the occurrence of arrhythmia events, since the P wave interval showed a negative correlation with the small HDL percentage, while it correlated positively with the large HDL percentage. In comparison with healthy subjects, Gaál et al. found decreased concentrations of all HDL subfractions in patients with systemic lupus erythematosus [48]. This highlights that our observation does not apply to each autoimmune disease.

In addition, the triglyceride-rich lipoprotein IDL percentage was significantly higher in SSc patients compared to the control group. In patients with multiple sclerosis, increased IDL levels and the IDL-C level are associated with increased cardiovascular risk and correlated with the elevated systolic blood pressure, respectively [49]. Liu et al. demonstrated that the elevated IDL levels are associated with the progression of carotid atherosclerosis [50].

We also demonstrated that sVCAM-1, sICAM-1 and serum PGRN levels showed significant negative correlation with a large HDL percentage. On the other hand, a small HDL percentage showed a positive correlation with the PGRN level. Nádró et al. also found a positive correlation between a small HDL percentage and the PGRN levels, and a negative correlation between a large HDL percentage and the PGRN in patients with familial hypercholesterinemia [51]. They assumed that the PGRN exerts its antiatherogenic effects by influencing the composition of the HDL.

Previously, lower mean LDL size was detected in patients with early rheumatoid arthritis compared to controls [52]. In our study, the mean LDL size was also lower in the SSc group, but the difference between the SSc and control groups did not reach the level of statistical significance.

The serum leptin level in SSc patients was higher, but the difference between the patients and controls (14.17 ng/mL vs. 9.81 ng/mL, *p* = 0.41) did not reach the level of significance, which is in agreement with previous results [27]. Of note, the serum leptin level of scleroderma patients showed a significant positive correlation with the maximal P wave interval, which can be related to the proinflammatory effect of the leptin [53]. Based on these observations, we can assume that leptin, due to its proinflammatory property, may exert an arrhythmogenic effect in SSc patients.

## 5. Conclusions

Atrial arrhythmias frequently occur in scleroderma patients compared to the general population [10]; thus, the recognition of high-risk patients is of particular importance. Based on our results, certain electrocardiographic and echocardiographic markers, together with the measurement of serum PGRN, sVCAM-1, sICAM-1, leptin, CRP, LDL-C, oxLDL, LDL and HDL subfractions, can provide valuable information regarding atrial arrhythmia risk in SSc patients.

Our results highlight that different lipid compositions among scleroderma patients and healthy controls correlate in an altered way with atrial electrocardiographic markers. This observation suggests that autoimmune inflammatory processes may differently influence the effects of lipids on atrial arrhythmia susceptibility in scleroderma patients. Further investigations are needed to elucidate these assumptions.

## Figures and Tables

**Table 1:** Inclusion and exclusion criteria for the study enrollment.

Inclusion Criteria	Exclusion Criteria
age above 18 years	existing diabetes mellitus
age under 70 years	presence of structural heart diseases
existing systemic sclerosis	arrhythmia events, especially atrial fibrillation in past medical history
euthyroid state	presence of untreated hypothyroidism
normal base blood pressure values	presence of untreated hypertension

**Table 2:** Basic clinical characteristics of the examined populations.

	SSc Patients (*n* = 26)	Control Group (*n* = 36)	*p* Values
ratio of female patients	20/26	25/36	n.s.
BMI	24.96 ± 4.93	28.11 ± 7.97	n.s.
age	56.82 ± 2.27	53.21 ± 3.61	n.s.
statins among medications	7/26	5/36	n.s.
	**Main Properties of Our SSc Population**		
type of SSc	12/26 lcSSc, 14/26 dcSSc		
time from diagnosis	4.1 ± 1.8 years		
time from first symptoms	6.5 ± 2.1 years		

In this table, we highlight the time elapsed from the diagnosis and the emergence of the first clinical symptoms to our examinations. BMI: body mass index, lcSSc: limited cutaneous scleroderma, dcSSc: diffuse cutaneous scleroderma, n.s.: not significant.

**Table 3:** Electrocardiographic and echocardiographic parameters of the examined populations. Data are presented as mean ± standard deviation.

Electrocardiographic Parameters	SSc Patients (*n* = 26)	Control Group (*n* = 36)	*p* Values
P wave interval (msec)	92.92 ± 18.2	69.11 ± 13.4	****
P max (msec)	113.5 ± 20.9	102.8 ± 15.3	*
P wave dispersion (msec)	44.08 ± 10	43.14 ± 9.6	n.s.
PP distance (msec)	910.61 ± 115.7	903.63 ± 143	n.s.
PQ interval (msec)	149.6 ± 25.37	142.3 ± 15.5	n.s.
**Echocardiographic** **Parameters**			
LA diameter (mm)	39.1 ± 4.5	37.2 ± 3.78	n.s.
LA area (cm^2^)	16.1 ± 4.6	13.2 ± 2.9	**
VCI (mm)	15.3 ± 2.13	14.1 ± 2.2	n.s.
mitral E/A (cm/sec)	1.6 ± 0.46	1.29 ± 0.38	n.s.
E/e’	12.39 ± 3.9	8.88 ± 1.9	****
TAPSE (mm)	26 ± 5.38	30 ± 3.8	***
RVP (mmHg)	19.8 ± 7	16.7 ± 4.38	*
EF (%)	56 ± 6.1	58.8 ± 5.7	n.s.
EDLVD (mm)	50.2 ± 11.6	48.8 ± 5	n.s.

LA: left atrium, VCI: inferior vena cava, E/e’: estimated left ventricular filling pressure, TAPSE: tricuspid annular plane systolic excursion, RVP: right ventricular pressure, EF: left ventricular ejection fraction, EDLVD: end-diastolic left ventricular diameter, * *p* < 0.05; ** *p* < 0.01; *** *p*< 0.001; **** *p* < 0.0001; n.s.: not significant.

**Table 4:** Summary of the laboratory parameters of the examined patients.

	SSc Patients	Control Group	*p* Values
CRP (mg/L)	5.04 ± 1.07	2.69 ± 0.96	*
HScTnT (ng/L)	17.8 (9.5–27.75)	8 (5–10)	**
CK (U/L)	101.65 ± 50.2	157.31 ± 85.7	*
NT-proBNP (ng/L)	120 (80–374)	43 (17–70)	****
sTSH (mU/L)	1.67 ± 0.43	1.97 ± 0.74	n.s.
fT3 (pmol/L)	4.78 ± 0.4	5.12 ± 1.51	n.s.
fT4 (pmol/L)	15.78 ± 2.62	15.47 ± 2	n.s.
sICAM-1 (ng/mL)	230.2 ± 76.4	186.4 ± 32.06	**
sVCAM-1 (ng/mL)	655.7 ± 109.14	586.2 ± 98.12	*
PGRN (ng/mL)	37 ± 9.09	36.3 ± 6.25	n.s.
TG (mmol/L)	1.47 ± 0.8	1.85 ± 1	n.s.
TC (mmol/L)	4.86 ± 0.95	5.59 ± 1.07	**
LDL-C (mmol/L)	2.99 ± 0.62	3.43 ± 0.77	*
HDL-C (mmol/L)	1.34 ± 0.29	1.39 ± 0.34	n.s.
Apo A-I (g/L)	1.41 ± 0.2	1.52 ± 0.22	*
Apo B (g/L)	0.99 ± 0.25	1.06 ± 0.25	n.s.
Lp(a) (mg/L)	64.5 (32.25–112.3)	65 (35–138)	n.s.
small HDL %	22.03 ± 7.2	23.33 ± 6.6	n.s.
intermediate HDL %	48.56 ± 4	50.54 ± 3.87	*
large HDL %	29.42 ± 8.9	26.14 ± 7.16	n.s.
IDL %	24.25 ± 3.8	19.39 ± 3.3	****
VLDL%	18.78 ± 2.9	20.02 ± 3.6	n.s.
large LDL %	28.25 ± 5.4	31.92 ± 4.45	**
small LDL %	1.89 ± 2.23	0.25 ± 0.39	****
mean LDL size (nm)	27.04 ± 0.03	26.81 ± 0.05	n.s.
oxLDL (U/L)	67.3 ± 18.72	74.54 ± 18.1	n.s.
leptin (ng/mL)	14.17 (5.64–31.53)	9. 8 (5.7–18.01)	n.s.

Data are presented as mean ± standard deviation or median and interquartile range. HScTnT: high sensitivity cardiac troponin T, NT-proBNP: N-terminal pro brain natriuretic protein, CK: creatine-kinase, CRP: C-reactive protein, sTSH: supersensitive thyroid stimulating hormone, fT3: free triiodothyronine, fT4: free thyroxine, TG: triglyceride, TC: total cholesterol, LDL-C: low-density lipoprotein cholesterol, HDL-C: high-density lipoprotein cholesterol, Lp (a): lipoprotein a, Apo A-I: apolipoprotein A-I, Apo B: apolipoprotein B, ox LDL: oxidized LDL, VLDL: very low-density lipoprotein, IDL: intermediate-density lipoprotein, * *p* < 0.05; ** *p* < 0.01; **** *p* < 0.0001; n.s.: not significant.

## Data Availability

All data will be made available on request.

## Supplementary Materials

The following supporting information can be downloaded at https://www.mdpi.com/article/10.3390/biomedicines13010220/s1, Table S1: Additional statistical data.

## References

[1] Geyer M., Muller-Ladner U. (2011). The pathogenesis of systemic sclerosis revisited. Clin. Rev. Allergy Immunol..

[2] Peoples C., Medsger T.A., Lucas M., Rosario B.L., Feghali-Bostwick C.A. (2016). Gender differences in systemic sclerosis: Relationship to clinical features, serologic status and outcomes. J. Scleroderma Relat. Disord..

[3] Tzelepis G.E., Kelekis N.L., Plastiras S.C., Mitseas P., Economopoulos N., Kampolis C., Gialafos E.J., Moyssakis I., Moutsopoulos H.M. (2007). Pattern and distribution of myocardial fibrosis in systemic sclerosis: A delayed enhanced magnetic resonance imaging study. Arthritis Rheum..

[4] Bruni C., Buch M.H., Furst D.E., De Luca G., Djokovic A., Dumitru R.B., Giollo A., Polovina M., Steelandt A., Bratis K. (2022). Primary systemic sclerosis heart involvement: A systematic literature review and preliminary data-driven, consensus-based WSF/HFA definition. J. Scleroderma Relat. Disord..

[5] Gawalko M., Balsam P., Lodzinski P., Grabowski M., Krzowski B., Opolski G., Kosiuk J. (2020). Cardiac Arrhythmias in Autoimmune Diseases. Circ. J..

[6] Fernandez-Codina A., Simeon-Aznar C.P., Pinal-Fernandez I., Rodriguez-Palomares J., Pizzi M.N., Hidalgo C.E., Guillen-Del Castillo A., Prado-Galbarro F.J., Sarria-Santamera A., Fonollosa-Pla V. (2017). Cardiac involvement in systemic sclerosis: Differences between clinical subsets and influence on survival. Rheumatol. Int..

[7] Bournia V.K., Tountas C., Protogerou A.D., Panopoulos S., Mavrogeni S., Sfikakis P.P. (2018). Update on assessment and management of primary cardiac involvement in systemic sclerosis. J. Scleroderma Relat. Disord..

[8] Tilly M.J., Geurts S., Zhu F., Bos M.M., Ikram M.A., de Maat M.P.M., de Groot N.M.S., Kavousi M. (2023). Autoimmune diseases and new-onset atrial fibrillation: A UK Biobank study. Europace.

[9] Vrancianu C.A., Gheorghiu A.M., Popa D.E., Chan J.S.K., Satti D.I., Lee Y.H.A., Hui J.M.H., Tse G., Ancuta I., Ciobanu A. (2022). Arrhythmias and Conduction Disturbances in Patients with Systemic Sclerosis—A Systematic Literature Review. Int. J. Mol. Sci..

[10] Fairley J.L., Ross L., Quinlivan A., Hansen D., Paratz E., Stevens W., Kistler P.M., McLellan A., La Gerche A., Nikpour M. (2023). Sudden cardiac death, arrhythmias and abnormal electrocardiography in systemic sclerosis: A systematic review and meta-analysis. Semin. Arthritis Rheum..

[11] Edigin E., Ojemolon P.E., Eseaton P.O., Shaka H., Akuna E., Asemota I.R., Manadan A. (2021). Systemic Sclerosis Is Associated With Increased Inpatient Mortality in Patients Admitted for Atrial Fibrillation: Analysis of the National Inpatient Sample. J. Clin. Rheumatol..

[12] Radwan Y.A., Kurmann R.D., Sandhu A.S., El-Am E.A., Crowson C.S., Matteson E.L., Osborn T.G., Warrington K.J., Mankad R., Makol A. (2021). Systemic Sclerosis Portends Increased Risk of Conduction and Rhythm Abnormalities at Diagnosis and During Disease Course: A US Population-Based Cohort. J. Scleroderma Relat. Disord..

[13] Aktoz M., Yilmaztepe M., Tatli E., Turan F.N., Umit E.G., Altun A. (2011). Assessment of ventricular and left atrial mechanical functions, atrial electromechanical delay and P wave dispersion in patients with scleroderma. Cardiol. J..

[14] Wokhlu N., Hsu V.M., Wilson A., Moreyra A.E., Shindler D. (2006). P-wave amplitude and pulmonary artery pressure in scleroderma. J. Electrocardiol..

[15] Sharifkazemi M., Nazarinia M., Arjangzade A., Goldust M., Hooshanginezhad Z. (2022). Diagnosis of Simultaneous Atrial and Ventricular Mechanical Performance in Patients with Systemic Sclerosis. Biology.

[16] Agoston G., Gargani L., Miglioranza M.H., Caputo M., Badano L.P., Moreo A., Muraru D., Mondillo S., Moggi Pignone A., Matucci Cerinic M. (2014). Left atrial dysfunction detected by speckle tracking in patients with systemic sclerosis. Cardiovasc. Ultrasound.

[17] Durmus E., Sunbul M., Tigen K., Kivrak T., Ozen G., Sari I., Direskeneli H., Basaran Y. (2015). Right ventricular and atrial functions in systemic sclerosis patients without pulmonary hypertension. Speckle-tracking echocardiographic study. Herz.

[18] Barsotti S.S.C., d’Ascanio A., Parma A., Emdin M., Conti U., Mosca M., Della Rossa A. (2017). High sensitivity troponin might be a marker of subclinical scleroderma heart involvement: A preliminary study. J. Scleroderma. Relat. Disord..

[19] Ferraz-Amaro I., Delgado-Frias E., Hernandez-Hernandez V., Sanchez-Perez H., de Armas-Rillo L., Armas-Gonzalez E., Machado J.D., Diaz-Gonzalez F. (2021). HDL cholesterol efflux capacity and lipid profile in patients with systemic sclerosis. Arthritis Res. Ther..

[20] Kotyla P.J., Gozdzik J., Lewicki M., Kotulska A.T., Kucharz E.J. (2006). Serum lipid profile in patients with systemic sclerosis: Relationship to the thyreometabolic state. Rheumatol. Int..

[21] Lippi G., Caramaschi P., Montagnana M., Salvagno G.L., Volpe A., Guidi G. (2006). Lipoprotein[a] and the lipid profile in patients with systemic sclerosis. Clin. Chim. Acta.

[22] Gonzalez-Gay M.A., Gonzalez-Juanatey C. (2014). Inflammation and lipid profile in rheumatoid arthritis: Bridging an apparent paradox. Ann. Rheum. Dis..

[23] Matsuura E., Kobayashi K., Inoue K., Lopez L.R., Shoenfeld Y. (2005). Oxidized LDL/beta2-glycoprotein I complexes: New aspects in atherosclerosis. Lupus.

[24] Rabquer B.J., Hou Y., Del Galdo F., Kenneth Haines G., Gerber M.L., Jimenez S.A., Seibold J.R., Koch A.E. (2009). The proadhesive phenotype of systemic sclerosis skin promotes myeloid cell adhesion via ICAM-1 and VCAM-1. Rheumatology.

[25] Denton C.P., Bickerstaff M.C., Shiwen X., Carulli M.T., Haskard D.O., Dubois R.M., Black C.M. (1995). Serial circulating adhesion molecule levels reflect disease severity in systemic sclerosis. Br. J. Rheumatol..

[26] Snarskaya E.S., Vasileva K.D. (2022). Localized scleroderma: Actual insights and new biomarkers. Int. J. Dermatol..

[27] Zhao J.H., Huang X.L., Duan Y., Wang Y.J., Chen S.Y., Wang J. (2017). Serum adipokines levels in patients with systemic sclerosis: A meta-analysis. Mod. Rheumatol..

[28] Lorincz H., Somodi S., Ratku B., Harangi M., Paragh G. (2023). Crucial Regulatory Role of Organokines in Relation to Metabolic Changes in Non-Diabetic Obesity. Metabolites.

[29] Michalska-Jakubus M., Sawicka K., Potembska E., Kowal M., Krasowska D. (2019). Clinical associations of serum leptin and leptin/adiponectin ratio in systemic sclerosis. Postep. Dermatol. Alergol..

[30] Ferri C., Valentini G., Cozzi F., Sebastiani M., Michelassi C., La Montagna G., Bullo A., Cazzato M., Tirri E., Storino F. (2002). Systemic sclerosis: Demographic, clinical, and serologic features and survival in 1,012 Italian patients. Medicine.

[31] Magnani J.W., Williamson M.A., Ellinor P.T., Monahan K.M., Benjamin E.J. (2009). P wave indices: Current status and future directions in epidemiology, clinical, and research applications. Circ. Arrhythm. Electrophysiol..

[32] Varro A., Baczko I. (2010). Possible mechanisms of sudden cardiac death in top athletes: A basic cardiac electrophysiological point of view. Pflug. Arch..

[33] Issa M.F., Yousry A., Tuboly G., Juhasz Z., AbuEl-Atta A.H., Selim M.M. (2023). Heartbeat classification based on single lead-II ECG using deep learning. Heliyon.

[34] Pall A., Czifra A., Sebestyen V., Becs G., Kun C., Balla J., Paragh G., Lorincz I., Pall D., Padra T.J. (2016). Hemodiafiltration and hemodialysis differently affect P wave duration and dispersion on the surface electrocardiogram. Int. Urol. Nephrol..

[35] Dilaveris P., Tousoulis D. (2017). P-wave dispersion measurement: Methodological considerations. Indian Pacing Electrophysiol. J..

[36] Juhasz I., Ujfalusi S., Seres I., Lorincz H., Varga V.E., Paragh G., Somodi S., Harangi M., Paragh G. (2022). Afamin Levels and Their Correlation with Oxidative and Lipid Parameters in Non-diabetic, Obese Patients. Biomolecules.

[37] Bielous-Wilk A., Poreba M., Staniszewska-Marszalek E., Poreba R., Podgorski M., Kalka D., Jagielski D., Rusiecki L., Pilecki W., Baran E. (2009). Electrocardiographic evaluation in patients with systemic scleroderma and without clinically evident heart disease. Ann. Noninvasive Electrocardiol..

[38] Nordin A., Bjornadal L., Larsson A., Svenungsson E., Jensen-Urstad K. (2014). Electrocardiography in 110 patients with systemic sclerosis: A cross-sectional comparison with population-based controls. Scand. J. Rheumatol..

[39] Sahn D.J., DeMaria A., Kisslo J., Weyman A. (1978). Recommendations regarding quantitation in M-mode echocardiography: Results of a survey of echocardiographic measurements. Circulation.

[40] Lester S.J., Ryan E.W., Schiller N.B., Foster E. (1999). Best method in clinical practice and in research studies to determine left atrial size. Am. J. Cardiol..

[41] Lang R.M., Badano L.P., Mor-Avi V., Afilalo J., Armstrong A., Ernande L., Flachskampf F.A., Foster E., Goldstein S.A., Kuznetsova T. (2015). Recommendations for cardiac chamber quantification by echocardiography in adults: An update from the American Society of Echocardiography and the European Association of Cardiovascular Imaging. Eur. Heart J. Cardiovasc. Imaging.

[42] Zhong X., Yu J., Zhao D., Teng J., Jiao H. (2023). Association between serum apolipoprotein A1 and atrial fibrillation in the Chinese population: A case-control study. BMC Cardiovasc. Disord..

[43] Malajczuk C.J., Gandhi N.S., Mancera R.L. (2021). Structure and intermolecular interactions in spheroidal high-density lipoprotein subpopulations. J. Struct. Biol. X.

[44] Huang C., Zhang J., Huang J., Li H., Wen K., Bao J., Wu X., Sun R., Abudukeremu A., Wang Y. (2023). Proteomic and functional analysis of HDL subclasses in humans and rats: A proof-of-concept study. Lipids Health Dis..

[45] Sacks F.M., Furtado J.D., Jensen M.K. (2022). Protein-based HDL subspecies: Rationale and association with cardiovascular disease, diabetes, stroke, and dementia. Biochim. Biophys. Acta Mol. Cell Biol. Lipids.

[46] Ding W.Y., Protty M.B., Davies I.G., Lip G.Y.H. (2022). Relationship between lipoproteins, thrombosis, and atrial fibrillation. Cardiovasc. Res..

[47] Szabo Z., Harangi M., Lorincz I., Seres I., Katona E., Karanyi Z., Paragh G. (2005). Effect of hyperlipidemia on QT dispersion in patients without ischemic heart disease. Can. J. Cardiol..

[48] Gaal K., Tarr T., Lorincz H., Borbas V., Seres I., Harangi M., Fulop P., Paragh G. (2016). High-density lipopoprotein antioxidant capacity, subpopulation distribution and paraoxonase-1 activity in patients with systemic lupus erythematosus. Lipids Health Dis..

[49] Radikova Z., Penesova A., Vlcek M., Havranova A., Sivakova M., Siarnik P., Zitnanova I., Imrich R., Kollar B., Turcani P. (2018). LDL and HDL lipoprotein subfractions in multiple sclerosis patients with decreased insulin sensitivity. Endocr. Regul..

[50] Liu T., Zhao D., Wang M., Sun J., Liu J., Li J., Duan Y., Sun Z., Hu P., Liu J. (2023). Association between Intermediate-Density Lipoprotein Particles and the Progression of Carotid Atherosclerosis: A Community-Based Cohort Study. J. Atheroscler. Thromb.

[51] Nadro B., Lorincz H., Juhasz L., Szentpeteri A., Sztanek F., Varga E., Pall D., Paragh G., Harangi M. (2022). Determination of Serum Progranulin in Patients with Untreated Familial Hypercholesterolemia. Biomedicines.

[52] Rizzo M., Spinas G.A., Cesur M., Ozbalkan Z., Rini G.B., Berneis K. (2009). Atherogenic lipoprotein phenotype and LDL size and subclasses in drug-naive patients with early rheumatoid arthritis. Atherosclerosis.

[53] Lorincz H., Ratku B., Csiha S., Seres I., Szabo Z., Paragh G., Harangi M., Somodi S. (2023). Impaired Organokine Regulation in Non-Diabetic Obese Subjects: Halfway to the Cardiometabolic Danger Zone. Int. J. Mol. Sci..

