# Supplementary material for: Inflammatory Biomarkers and Lipid Parameters May Predict an Increased Risk for Atrial Arrhythmias in Patients with Systemic Sclerosis
Source: Biomedicines. 2025 Jan 16;13(1):220. doi: 10.3390/biomedicines13010220 (PMC11762971; doi:10.3390/biomedicines13010220)
Supplement: Supplementary file 1 [file biomedicines-13-00220-s001.zip › biomedicines-3426083-supplementary.pdf]

Table S1: Additional statistical data.

P max	control	SSc
Descriptive statistics		
Minimum	70,00	78,00
25% Percentile	91,00	99,50
Median	101,5	117,0
75% Percentile	114,0	126,5
Maximum	134,0	180,0
Range	64,00	102,0
Mean	102,8	113,5
Std. Deviation	15,33	21,95
Std. Error of Mean	2,554	4,304
Lower 95% CI of mean	97,56	104,6
Upper 95% CI of mean	107,9	122,4
Kolmogorov-Smirnov test		
KS distance	0,06404	0,1390
P value	>0,1000	>0,1000
Passed normality test (alpha=0.05)?	Yes	Yes
P value summary	ns	ns
**E/e’**	**control**	**SSc**
Descriptive statistics		
Minimum	5,696	6,480
25% Percentile	7,447	9,583
Median	8,922	11,72
75% Percentile	10,28	14,13
Maximum	12,60	21,42
Range	6,907	14,94
Mean	8,882	12,39
Std. Deviation	1,880	3,918
Std. Error of Mean	0,3011	0,7683
Lower 95% CI of mean	8,273	10,80
Upper 95% CI of mean	9,492	13,97
Kolmogorov-Smirnov test		
KS distance	0,1085	0,1810
P value	>0,1000	>0,1000
Passed normality test (alpha=0.05)?	Yes	Yes
P value summary	ns	ns
**TAPSE**	**control**	**SSc**
Descriptive statistics		
Minimum	23,00	18,00
25% Percentile	27,00	22,00
Median	30,00	26,00
75% Percentile	33,00	29,75
Maximum	39,00	37,00
Range	16,00	19,00
Mean	30,18	26,12
Std. Deviation	3,797	5,376
Std. Error of Mean	0,6079	1,054
Lower 95% CI of mean	28,95	23,94
Upper 95% CI of mean	31,41	28,29
Kolmogorov-Smirnov test		
KS distance	0,1068	0,1239
P value	>0,1000	>0,1000
Passed normality test (alpha=0.05)?	Yes	Yes
P value summary	ns	ns
